# Causal relationship between inflammatory factors and cerebral small vessel disease: Univariate, multivariate, and summary‐data‐based mendelian randomization analysis

**DOI:** 10.1002/brb3.3399

**Published:** 2024-02-10

**Authors:** Tian‐Ci Qiao, Hao‐Yu Tian, Shi‐Zhe Shan, Li‐Li Shan, Zheng‐Yu Peng, Jia Ke, Meng‐Ting Li, Yang Wu, Yan Han

**Affiliations:** ^1^ Department of Neurology Yueyang Hospital of Integrated Traditional Chinese and Western Medicine Shanghai China; ^2^ Shanghai University of Traditional Chinese Medicine Shanghai China; ^3^ Guang'anmen Hospital China Academy of Chinese Medical Sciences Beijing China; ^4^ Taihe Hospital Hubei University of Medicine Shiyan Hubei China

**Keywords:** cerebral small vessel disease, inflammatory factors, mendelian randomization, summary‐data‐based mendelian randomization

## Abstract

**Objective:**

To explore the impact of inflammatory factors on the incidence of cerebral small vessel disease (CSVD), we performed a mendelian randomization (MR) study to analyze the causal relationship between multiple inflammatory factors and CSVD imaging markers and utilized summary‐data‐based mendelian randomization (SMR) analysis to infer whether the impact of instrumental variables (IVs) on disease is mediated by gene expression or DNA methylation.

**Methods:**

Using public databases such as UKB and IEU, and original genome‐wide association studies, we obtained IVs related to exposure (inflammatory factors) and outcome (CSVD imaging markers). We performed the inverse variance weighted, weighted median, and MR‐Egger methods to assess causal effects between exposure and outcome in univariate MR analysis. To evaluate their heterogeneity, a series of sensitivity analyses were conducted, including the Cochrane Q test, MR‐Egger intercept test, MR‐Presso, and leave‐one‐out analysis. We also applied mediation and multivariate MR analysis to explore the interactions between positive exposures on the same outcome. Additionally, we conducted the SMR, which utilizes instruments within or near relevant genes in blood or brain tissues, to elucidate the causal associations with CSVD markers.

**Results:**

ABO Univariate MR of multiple cohorts revealed that the risk of small vessel stroke (SVS) increases with elevated levels of TNF‐related apoptosis‐inducing ligand (TRAIL, OR, 1.23, 95% CI, 1.08–1.39) and interleukin‐1 receptor‐like 2, (IL‐1RL2, OR, 1.29, 95% CI, 1.04–1.61). IL‐18 was a potential risk factor for extensive basal ganglia perivascular space burden (BGPVS, OR, 1.02, 95% CI, 1.00–1.05). Moreover, the risk of extensive white matter perivascular space burden (WMPVS) decreased with rising levels of E‐selectin (OR, .98, 95% CI, .97–1.00), IL‐1RL2 (OR, .97, 95% CI, .95–1.00), IL‐3 receptor subunit alpha (IL‐3Ra, OR, .98, 95% CI, .97–1.00), and IL‐5 receptor subunit alpha (IL‐5Ra, OR, .98, 95% CI, .97–1.00). Mediation and multivariate MR analysis indicated that E‐selectin and IL‐3Ra might interact during the pathogenesis of WMPVS. SMR estimates showed that TRAIL‐related IVs rs5030044 and rs2304456 increased the risk of SVS by increasing the expression of gene Kininogen‐1 (KNG1) in the cerebral cortex, particularly in the frontal cortex (*β*smr = .10, *P*smr = .003, FDR = .04). Instruments (rs507666 and rs2519093) related to E‐selectin and IL‐3Ra could increase the risk of WMPVS by enhancing DNA methylation of the gene ABO in blood tissue (*β*smr = .01–.02, *P*smr = .001, FDR = .01–.03).

**Conclusion:**

According to MR and SMR analysis, higher levels of TRAIL increased the risk of SVS by upregulating gene expression of KNG1 in brain cortex tissues. In addition, protective effects of E‐selectin and IL‐3a levels on WMPVS were regulated by increased DNA methylation of gene ABO in blood tissue.

## 1 INTRODUCTION

Cerebral small vessel disease (CSVD) refers to a group of clinical, imaging, and pathological syndromes caused by various cerebral vascular lesions involving small arteries, capillaries, and small veins (with diameters less than 400 μm) including five imaging markers, such as white matter hyperintensities (WMH), acute small vessel stroke (SVS), lacunar infarcts, cerebral microbleeds (CMBs), enlarged perivascular spaces (EPVS), and brain atrophy (Wardlaw et al., 2019). Cortical superficial siderosis and cortical cerebral microinfarct are the new markers of CSVD according to the Standards for Reporting Vascular Changes on Neuroimaging 2023 (Duering et al., 2023). Notably, the incidence of CSVD increases with age greatly, which is about 80% in people over 60 years old (Litak et al., 2020) and reaches 100% in those over 90 years old (Cannistraro et al., 2019). Clinical presentation of CSVD varies significantly, with 20% of elderly patients remaining asymptomatic, whereas others experience cognitive impairment, emotional, gait, or urinary disorders (Hussein & Anderson, 2021). CSVD significantly elevates the risk of recurrent ischemic stroke and vascular dementia (Hussein & Anderson, 2021), leading to a decline in the ability of daily life in patients and imposing heavy burdens on families and societies (Han et al., 2018). Recently, the emerging role of inflammation in the development of CSVD has become a focal point of research. To explore the potential causal relationship between inflammatory factors and CSVD, we performed this mendelian randomization (MR) study at the genetic level. MR is increasingly used to infer reliable causal relationships between risk factors and outcome diseases (Richmond & Smith, 2022). This approach, based on the random allocation of genetic variation during meiosis (Burgess & Thompson, 2015), uses instrumental variables (IVs) associated with exposures to assess the relationship between exposure and outcome. The random allocation of single nucleotide polymorphisms (SNPs) in offspring simulates the randomized controlled trial (RCT), which avoids the substantial consumption of time, manpower, and financial resources required for large‐scale RCTs, and MR analysis can effectively exclude confounding factors and identify the causal relationship between exposures and outcomes (Richmond & Smith, 2022).

## 2 METHODS

### 2.1 Study design

In this study, we performed univariate, multivariate MR, and mediation analysis. Bidirectional analysis was conducted in the univariate MR, using IVs to evaluate the genetic causal effects between multiple inflammatory factors on CSVD.

Sources of instruments: Genome‐wide association studies (GWAS) summary data of inflammatory factors were obtained from public databases: IEU and EBI, Table S1. SVS is identified as new small infarcts with high signals on the diffusion‐weighted imaging sequence and lacunes with diameters less than 15 mm according to the TOAST classification (Chen et al., 2012). WMH is diagnosed based on the T2‐FLAIR sequence. Fractional anisotropy (FA) and mean diffusivity (MD), derived from the diffusion tensor imaging sequence, MD primarily reflects the degree of diffusion of water molecules in the extracellular tissue, whereas FA reflects the integrity of cellular structures. Both are sensitive indicators for assessing white matter integrity (Alexander et al., 2007). CMBs are diagnosed by susceptibility weighted or T2‐weighted MRI sequence and categorized into deep and lobar CMBs based on their locations. Perivascular spaces (PVS), the physiological anatomical structure situated between cerebral small vessels and subarachnoid space, could be identified as visible on the T2‐weighted MRI sequence when there is abnormal clearance and accumulation of metabolic products and toxins in cerebrospinal fluid. The genome data for CSVD markers were obtained from the EBI public database and original GWAS (Duperron et al., 2023; Knol et al., 2020; Malik et al., 2018; Persyn et al., 2020; Traylor et al., 2021), Table S2.

IVs of exposure must satisfy three following assumptions: (1) IVs are significantly associated with exposure (*p* < 5e − 08) (Palmer et al., 2012); (2) the IVs should not be related to other confounding factors; (3) IVs are expected to affect the outcome exclusively through the exposure. Furthermore, to avoid vertical pleiotropy due to physical proximity on chromosomes, which can introduce endogeneity, IVs were corrected for intrinsic linkage disequilibrium before analysis, based on PLINK Clump data, with requirements set at *R^2^* < .001. SNPs strongly related to outcomes and confounding factors (*p* < 1e − 05) were identified and excluded using the PhenoScanner database. IVs with a minor allele frequency <1% were removed. To reduce the risk of Type II errors, IVs with an *F* < 10 were also excluded.

### 2.2 MR estimates

We utilized the TwoSampleMR R package for MR analysis to assess the genetic effects between inflammation factors and CSVD markers. For exposure with multiple IVs, we applied standard inverse variance weighted (IVW) (Burgess et al., 2015) as the main method in univariate MR, MR‐Egger regression (Bowden et al., 2015), and weighted median (Bowden et al., 2016) methods are complements to IVW, which can be used to assess the robustness of the MR results. For phenotypes associated with only one SNPs, the Wald Ratio method is employed to estimate the effect size from the exposure to the outcome. The Wald Ratio method utilizes the regression coefficients between the outcome and the exposure, along with the Wald ratio, to calculate the primary effect in single IVs regression. Additionally, bidirectional MR analysis was performed to estimate the potential reverse causal relationship, and the two‐step MR mediation analysis was conducted to explore the possible pathways between multiple exposures and outcomes. Multivariate MR analysis is an extension of univariate MR and could detect the joint causal effect of multiple exposures on a single outcome (Sanderson, 2021, Sanderson et al., 2019). All MR analyses were conducted in R software (version 4.3.1).

### 2.3 Sensitivity analysis

The MR‐Presso (Verbanck et al., 2018) and MR‐Egger intercept tests were used to identify potential horizontal pleiotropy by detecting outliers among the IVs. The Cochran's Q test and its *p*‐value are used to estimate the heterogeneity in univariate MR. MR‐Presso is needed to identify and ascertain which outlier impacts the direction of the outcome. To further elucidate whether the causal relationship is driven by any single SNP, we conducted the leave‐one‐out analysis: Each SNP associated with the exposure was successively omitted and IVW was repeated to determine whether the estimates remain significant effects. All the details of significant MR estimates are listed in Table S3. The scatter plots, funnel plots, and leave‐one‐out plots of significant MR estimates were exhibited in Figures S1–S17.

### 2.4 SMR analysis

Summary‐data‐based mendelian randomization (SMR) is a method that associates gene expression and DNA methylation levels with traits of interest, using GWAS summary data and quantitative trait loci (QTL) data (Zhu et al., 2016). SMR software (Linux version 1.3.1) was obtained from the official website (https://yanglab.westlake.edu.cn/software/smr/). This analysis utilized multiple gene expression QTL (eQTL) and DNA methylation QTL (mQTL) datasets, including GTEx V8 blood (*n* = 670) and seven brain region eQTL datasets (*n* = 129–194) (GTEx Consortium, 2020), LBC_BSGS blood mQTL dataset (*n* = 1980) (McRae et al., 2018; Wu et al., 2018), and the Brain‐mMeta mQTL dataset from Qi et al. (2018). Relevant genes and consequences of significant SNPs were searched on original GWAS studies and the NCBI database, and we conducted SMR analysis by using exposure‐related QTL data and CSVD GWAS data. With FDR value less than .05 was selected, and the HEIDI test *p*‐value over .01, which required no significant heterogeneity relationship. This step is crucial to determine whether the relationship between the target gene and the disease phenotype is causal or pleiotropic. The type of QTL is critical in determining whether the exposure affects the disease phenotype through gene eQTL or DNA mQTL.

### 2.5 Ethics and privacy statement

The data used in this study were sourced from publicly available GWAS summary data. These studies have been approved by ethical committees and undergone peer review and are published in accessible journals. The GWAS summary data do not contain any personal information or other potentially sensitive content; thereby, a secondary review by an ethics committee is not needed. This study followed the guidelines for MR studies as outlined by Strengthening the Reporting of Observational Studies in Epidemiology for Mendelian Randomization Studies (Skrivankova et al., 2021). Details were exhibited in the Supporting Information section.

## 3 RESULTS

### 3.1 MR estimates

**FIGURE 1:**
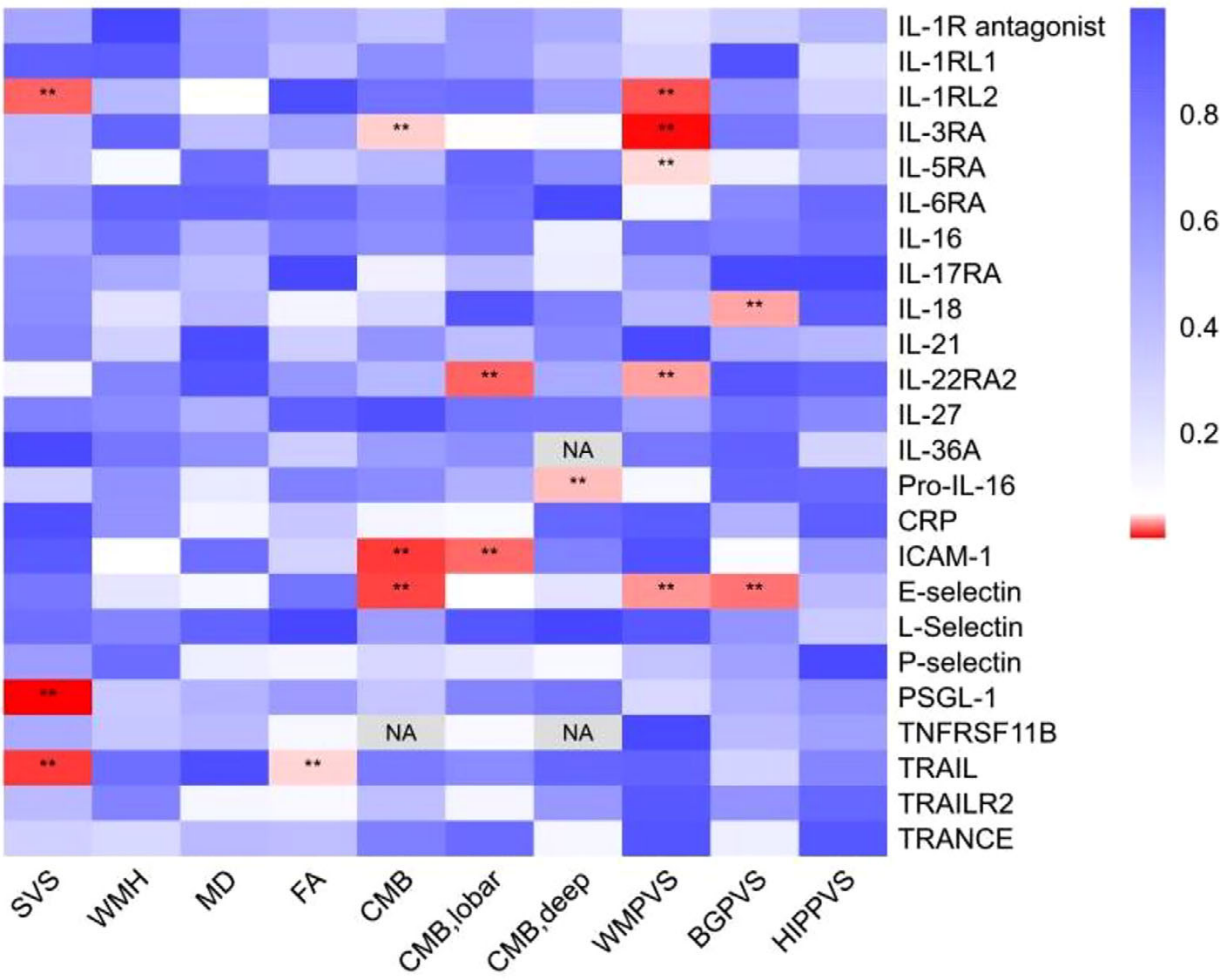
Causal relationship between serum inflammatory factors and CSVD markers. BGPVS, extensive basal ganglia perivascular space burden; CMB, cerebral microbleeds; FA, fractional anisotropy; HIPPVS, extensive hippocampal perivascular space burden; MD, mean diffusivity; SVS, small vessel stroke; WMH, log white matter hyperintensities volume; WMPVS, extensive white matter perivascular space burden.

**FIGURE 2:**
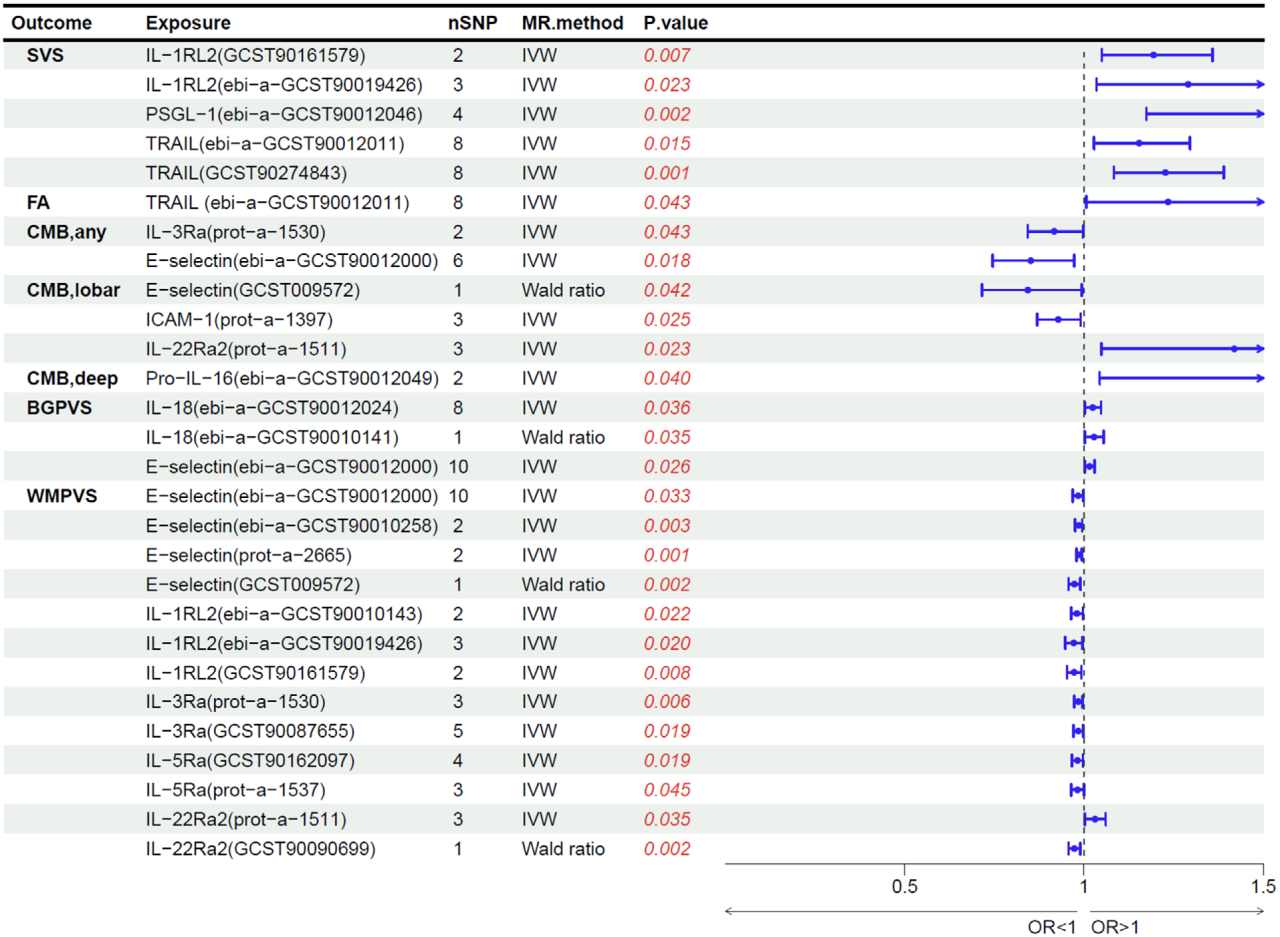
Forest plot of significant mendelian randomization (MR) estimates between inflammatory factors and cerebral small vessel disease (CSVD) markers.

The univariate MR revealed significant causal relationships between some inflammatory factors (TRAIL, E‐selectin, PSGL‐1, and certain interleukin (IL)‐related proteins) and the risk of CSVD imaging markers, Table 3 and Figures 1‐2. No significant reverse causal relationships were observed in univariate MR. Among the observed inflammatory factors, no significant risk or protective factors were found for WMH, MD, and hippocampal EPVS. For SVS, TRAIL (OR, 1.15, 95% CI, 1.03−1.30), PSGL‐1 (OR, 1.56, 95% CI, 1.17−2.08), and IL‐1 receptor‐like 2 (IL‐1RL2) (OR, 1.29, 95% CI, 1.04−1.61) showed significant causal effects. Additionally, TRAIL has a positive causal effect on FA values (β, .21, 95% CI, .01‐.41). Levels of E‐selectin (OR, 1.02, 95% CI, 1.00−1.03) and IL‐18 (OR, 1.02, 95% CI, 1.00−1.05) emerged as significant risk factors for extensive basal ganglia perivascular space burden (BGPVS). Several inflammatory proteins have significant causal effects on extensive white matter perivascular space burden (WMPVS), including the level of E‐selectin (OR, .98, 95% CI, .97–1.00), IL‐1 receptor‐like 2 (IL‐1RL2, OR, .97, 95% CI, .95–1.00), IL‐22 receptor subunit alpha‐2 (IL‐22Ra2, OR, 1.03, 95% CI, 1.00–1.06), IL‐3 receptor subunit alpha (IL‐3Ra, OR, .98, 95% CI, .97–1.00), and IL‐5 receptor subunit alpha (IL‐5Ra, OR, .98, 95% CI, .97–1.00). Diverse causal relationships of inflammatory proteins varied for different locations of CMBs: For CMBs in any brain regions, E‐selectin (OR, .85, 95% CI, .75–.97) and IL3Ra (OR, .92, 95% CI, .84–1.00) were the protective factors. E‐selectin (OR, .84, 95% CI, .72–.99), intercellular adhesion molecule 1 (ICAM‐1, OR, .93, 95% CI, .87–.99), and IL‐22Ra2 (OR, 1.42, 95% CI, 1.05–1.92) had potential causal associations with CMBs in brain lobes. Pro‐IL‐16 (OR, 2.43, 95% CI, 1.04–5.66) was the potential risk factor for CMBs in the deep brain region. The causal relationships of TRAIL and IL‐1RL2 with SVS, E‐selectin, IL‐1RL2, IL3Ra, and IL5Ra with WMPVS, and IL‐18 with BGPVS have been validated across different GWAS cohorts. Detailed univariate MR findings were presented in Table S3 and relevant SNPs were listed in Table S4.

Given that there were significant causal relationships between multiple inflammatory factor exposures and the same outcome, mediation analysis was performed for significant exposures in pairs. It was found that TRAIL levels have a positive causal effect on PSGL‐1 levels (β, .05, 95% CI, 2.5e−05–.11). However, PSGL‐1 did not demonstrate a significant mediating effect in the process by which TRAIL affects SVS, as detailed in Table 1. This suggested that PSGL‐1 and TRAIL might have independent mechanisms in affecting the onset of SVS. Additionally, as for the exposure of WMPVS, there was a significant causal relationship between *E*‐selectin levels and IL‐3Ra (β, .77, 95% CI, .50–1.04). However, due to the significant heterogeneity and pleiotropy, mediation analysis was not conducted. Further multivariate MR analysis was performed among positive inflammatory factor exposures. Some causal associations turned insignificant after adjusting some inflammatory factors as covariates, Table 2. Regarding WMPVS, after controlling for IL‐3Ra levels, the protective effect of *E*‐selectin lost its statistical significance (*p* = .46), and the protective effect of IL‐3Ra lost its statistical significance as well (*p* = .62). However, the level of TRAIL and PSGL‐1 still exhibited as the risk factors for SVS (*p* < .05) in multivariate MR analysis, which also demonstrated the independent mechanism in the development of SVS.

**TABLE 1:** Mediation analysis of inflammation factors on cerebral small vessel disease (CSVD) markers.

Outcome	Mediator	Exposure	Total effect	Direct effect	Mediation effect	
			(95%CI)	(95%CI)	(95%CI)	*p*
SVS	PSGL‐1	TRAIL	.14 (.03–.26)	.12 (.00–.23)	.02 (−.01–.05)	.11

Abbreviations: PSGL‐1, P‐selectin glycoprotein ligand 1 levels; SVS, small vessel stroke; TRAIL, TNF‐related apoptosis‐inducing ligand levels.

**TABLE 2:** Multivariable mendelian randomization estimates of inflammation factors and cerebral small vessel disease (CSVD) markers.

Exposure	Outcome	nsnp	Beta	SE	*p*	OR 95%CI
TRAIL	SVS	8	.12	.06	.047	1.13 (1.00–1.28)
PSGL‐1	SVS	4	.40	.15	.01	1.49 (1.11–2.00)
E‐selectin	WMPVS	10	−.01	.01	.46	.99 (.96–1.02)
IL‐3Ra	WMPVS	3	−.01	.02	.62	.99 (.96–1.02)

Abbreviations: IL‐3Ra, interleukin‐3 receptor subunit alpha; PSGL‐1, P‐selectin glycoprotein ligand 1 levels; SVS, small vessel stroke; TRAIL, TNF‐related apoptosis‐inducing ligand levels; WMPVS, extensive white matter perivascular space burden.

**TABLE 3:** Probes identified in the summary‐data‐based mendelian randomization (SMR) analysis between inflammatory factors and cerebral small vessel disease (CSVD) markers.

Phenotype	probeID	Gene	p_GWAS	p_e/mQTL	b_SMR	se_SMR	p_SMR	p_HEIDI	FDR	Tissue
WMPVS	cg11879188	ABO	0.0153	2.14E − 183	0.0134683	0.00419478	0.001323932	0.2203529	0.040743242	Whole blood
WMPVS	cg21160290	ABO	0.01522	0	0.00938044	0.00290816	0.001257282	0.2390162	0.040743242	Whole blood
WMPVS	cg22535403	ABO	0.01522	0	0.00960344	0.00297806	0.0012609	0.2143651	0.040743242	Whole blood
WMPVS	cg13506600	ABO	0.01132	1.53E − 79	0.0191036	0.00586342	0.00112164	0.1607792	0.040743242	Whole blood
WMPVS	cg24267699	ABO	0.00153	1.52E − 162	0.0138931	0.00433056	0.001335844	0.3683463	0.040743242	Whole blood
SVS	ENSG00000113889	KNG1	0.00001883	4.15E − 20	0.109561	0.037201	0.003228399	0.4856058	0.025827192	Barin cortex
SVS	ENSG00000113889	KNG1	0.00001565	2.11E − 21	0.0988105	0.0329106	0.002678636	0.5072561	0.01339318	Frontal cortex

Abbreviations: ABO, alpha 1‐3‐*N*‐acetylgalactosaminyltransferase and alpha 1‐3‐galactosyltransferase; GWAS, genome‐wide association studies; HEIDI, heterogeneity in dependent instruments; KNG1, kininogen 1; mQTL, DNA methylation quantitative trait loci; QTL, quantitative trait loci; SVS, small vessel stroke; WMPVS, extensive white matter perivascular space burden.

### 3.2 Sensitivity analysis

To evaluate the robustness of these MR results, a series of sensitivity analyses were conducted, including Cochran's *Q* test, MR‐Egger intercept test, and MR‐Presso global test. The univariate MR estimates showed that there was no significant horizontal pleiotropy or heterogeneity in these significant MR results, indicating robust findings.

### 3.3 SMR analysis

To prioritize genes with functional significance, SMR analysis was conducted using 10 eQTL and mQTL datasets on outcomes from the MR with significant causal relationships (SVS, WMPVS, BGPVS, FA, CMBs in any brain region). This analysis identified seven associations involving two unique genes (ABO, Kininogen‐1 (KNG1)), Figure 3. However, none of these genes were involved in both eQTL and mQTL datasets simultaneously. The SMR analysis revealed significant causal associations in the whole brain cortex and frontal cortex tissues. Probe ENSG00000113889, which tagged gene KNG1, showed a significant association between gene expression of KNG1 and the GWAS data of SVS (*β*smr = .10, *P*smr = .003). KNG1 is the associated gene for significant SNPs related to TRAIL levels (cis‐eQTL‐rs5030044: intron variant and cis‐eQTL‐rs2304456: missense variant). In blood tissue, five DNAm probes (cg11879188, cg21160290, cg22535403, cg13506600, and cg24267699) tagged gene ABO showed a significant relationship with WMPVS. Gene ABO is related to significant SNPs of E‐selectin (rs115478735, rs507666, rs2519093: intron variant), IL‐22Ra2 (rs507666: intron variant), and IL‐3Ra (rs507666, rs2519093: intron variant). These results remained statistically significant after correction for multiple testing, Tables S5–S8. These probes did not show a significant association with other phenotype of CSVD markers. The HEIDI test *p*‐values in the SMR were all over .01, suggesting no pleiotropic associations between these specific probes and CSVD markers.

FIGURE 3Summary‐data‐based mendelian randomization (SMR) locus plots. (a) Prioritizing genes around KNG1 in association with small vessel stroke in brain cortex tissue, (b) prioritizing genes around KNG1 in association with small vessel stroke in brain frontal cortex tissue. Top plot, gray dots represent the −log10 (*p* values) for single nucleotide polymorphisms (SNPs) from the GWAS of SVS, and rhombuses represent the −log10 (*p* values) for probes from the SMR test with solid rhombuses indicating that the probes pass HEIDI test and hollow rhombuses indicating that the probes do not pass the HEIDI test. Middle plot, eQTL results in blood for one probe, ENSG00000113889 probe, tagging KNG1. Bottom plot, location of gene tagged by the probes. Highlighted in maroon indicates probes that pass SMR threshold. (c) Prioritizing genes around ABO in association with extensive white matter perivascular space burden in blood tissue. Top plot, gray dots represent the −log10 (*p* values) for SNPs from the GWAS of WMPVS, and rhombuses represent the −log10 (*p* values) for probes from the SMR test with solid rhombuses indicating that the probes pass HEIDI test and hollow rhombuses indicating that the probes do not pass the HEIDI test. Middle plot, mQTL results in blood for two probes, cg11879188, cg21160290, cg22535403, cg13506600, and cg24267699 probes, tagging ABO. Bottom plot, location of gene tagged by the probes. Highlighted in maroon indicates probes that pass SMR threshold. ABO, alpha 1‐3‐*N*‐acetylgalactosaminyltransferase and alpha 1‐3‐galactosyltransferase; eQTL, expression quantitative trait loci; GWAS, genome‐wide association studies; HEIDI, heterogeneity in dependent instruments; KNG1, kininogen 1; mQTL, DNA methylation quantitative trait loci; SVS, small vessel stroke; SMR, summary data‐based mendelian randomization; WMPVS, white matter perivascular space burden.

**Figure:**
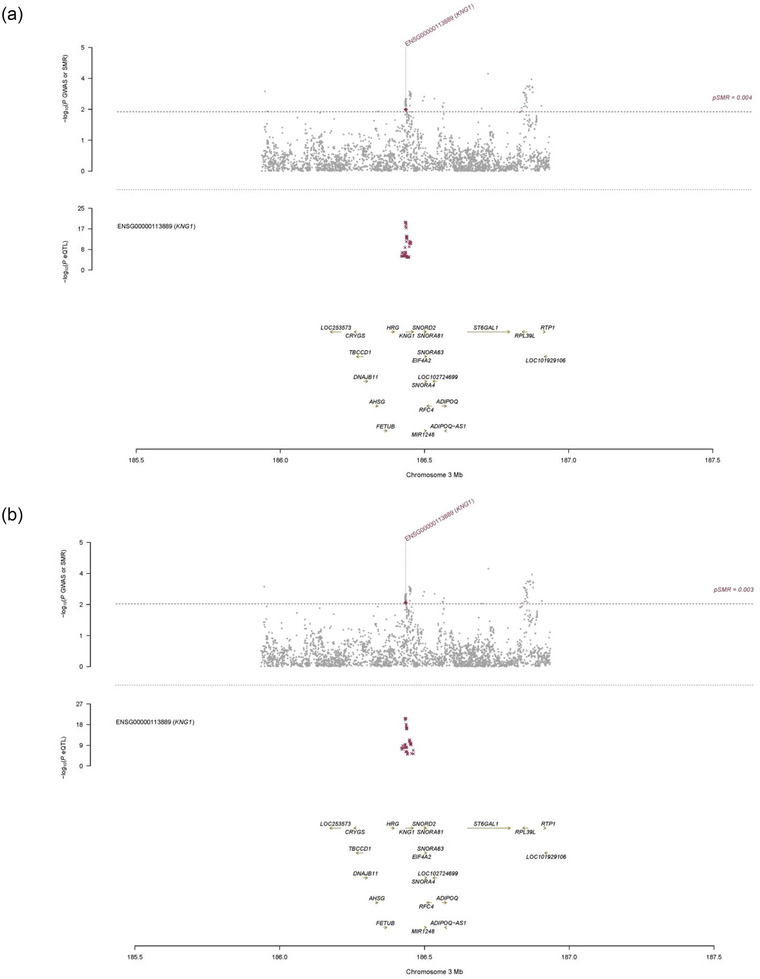

**Figure:**
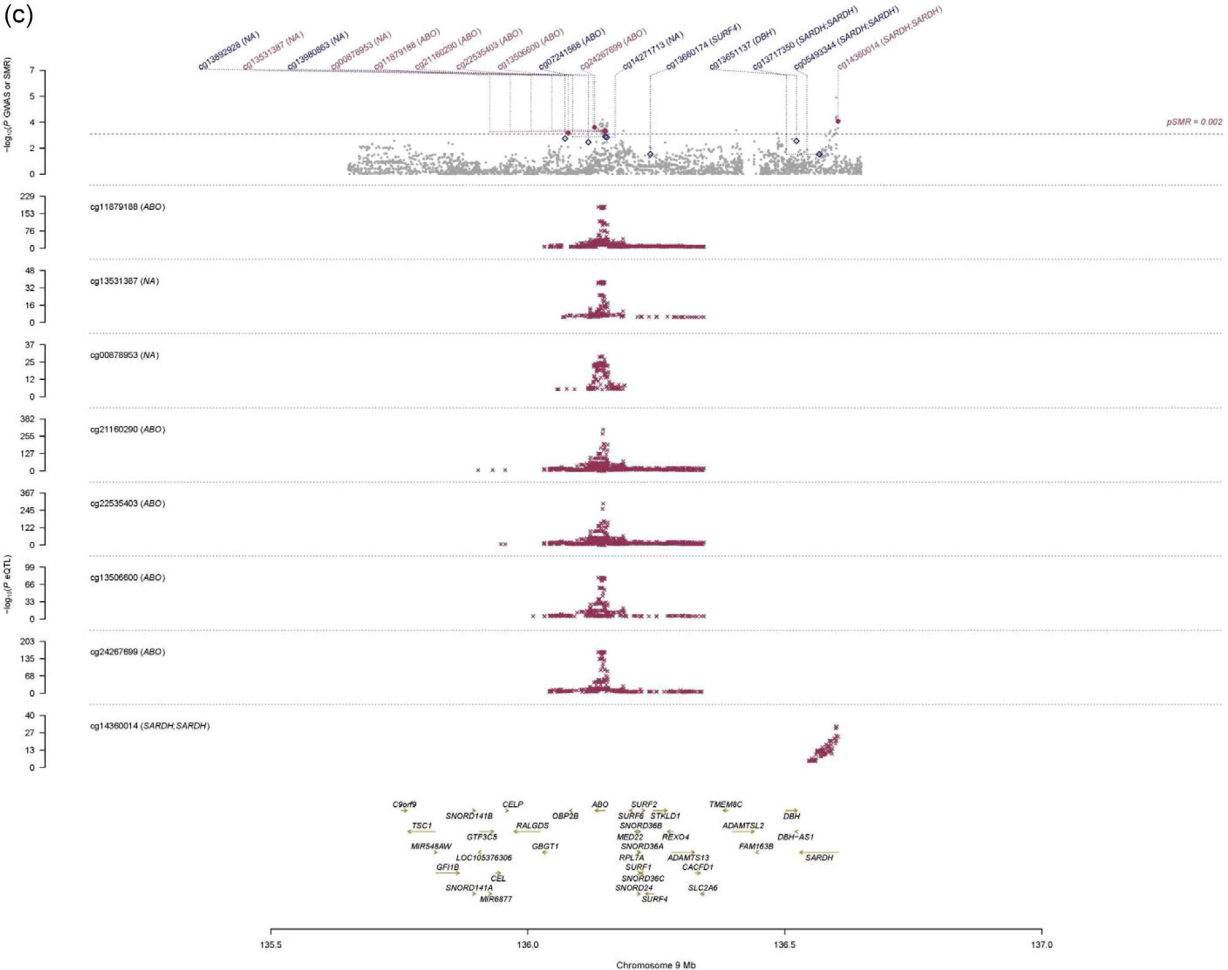

## 4 DISCUSSION

The leading cause of CSVD is commonly attributed to small artery sclerosis, which is commonly associated with aging, hypertension, and other vascular risk factors (Markus & de Leeuw, 2023). The pathogenesis of this CSVD type is thought to be multifactorial, involving a spectrum of mechanisms, such as microvascular lesions, endothelial cell dysfunction, blood–brain barrier disruption, and neuroinflammation (Hannawi, 2023). Previous cross‐sectional studies have demonstrated that multiple inflammatory biomarkers were associated with CSVD, including neutrophil count, tumor necrosis factor receptor 2, ICAM‐1, vascular cell adhesion molecule 1, total homocysteine, and other indicators (Jiang et al., 2022; Shoamanesh et al., 2015; Zhang et al., 2022). Our study aimed to explore the causal relationship between inflammatory factors and CSVD and attempted to elucidate the genetic mechanism by integrating GWAS, eQTL, and mQTL from various datasets. Univariate MR results indicated that high TRAIL levels were associated with an increased risk of SVS (OR, 1.15, 95% CI, 1.03–1.30), and this relationship was confirmed in another European cohort. Further SMR analysis revealed that two related SNPs, cis‐eQTL‐rs5030044 (intron variant) and cis‐eQTL‐rs2304456 (missense variant), were associated with the expression of gene KNG1 in the whole brain cortex and the frontal cortex, and the KNG1 gene expression was significantly associated with the risk of SVS after multiple corrections (*β*smr = .10, *P*smr = .003). This suggested that elevated levels of TRAIL may increase the risk of SVS by enhancing KNG1 gene expression in the cerebral cortex, particularly in the frontal lobe, through related SNPs.

TRAIL is a type II membrane protein on cell surface that can induce apoptosis by blocking G1–S progression (Qin et al., 2021). TRAIL is expressed in the cerebellum, spinal cord, glial cells, and vascular smooth muscle cells, but its expression level is relatively low in the healthy human brain (Forde et al., 2016, Qin et al., 2021). TRAIL signaling downstream of TLR7 activation may mediate neuronal apoptosis and could be associated with neurological diseases, including ischemic stroke, Alzheimer's disease, and multiple sclerosis (Qin et al., 2021). However, the role of TRAIL in vascular endothelial cells remains controversial. Some studies suggested that TRAIL might protect vascular endothelium during the development of arteriosclerosis, whereas others have found that TRAIL could induce apoptosis in human microvascular endothelial cells (Pritzker et al., 2004) and increase the permeability of vascular wall and the penetration of lipids and inflammatory cells (Kavurma et al., 2008), which could accelerate the process of atherosclerosis (Forde et al., 2016). KNG1 has recently been identified as a biomarker for diseases, such as colorectal cancer, oral cancer, gliomas, and many others. It can inhibit angiogenesis and metastasis, making it a potential target for future research (Xu et al., 2018). However, there has been no prior research on the roles of TRAIL protein levels and KNG1 gene expression in the pathogenesis of SVS. Our study revealed that elevated levels of TRAIL might increase the risk of SVS, and this occurs by increasing KNG1 expression through related IVs (cis‐eQTL‐rs5030044: intron variant and cis‐eQTL‐rs2304456: missense variant). and the mechanisms through which they affect the pathogenesis of SVS require further research.

The results of univariate MR analysis indicated that elevated levels of E‐selectin, IL‐3Ra, IL‐5Ra, and IL‐22R2 were significantly causally related to WMPVS. Among these, E‐selectin, IL‐3Ra, and IL‐5Ra were robust risk factors that have been validated in more than one GWAS cohort, whereas IL‐22Ra2 in two cohorts showed opposite results. To avoid Type I errors, further analysis and discussion were not performed for IL‐22Ra2. Although we found a significant causal relationship between E‐selectin and IL3Ra in two‐sample MR, further mediation analysis was not conducted due to their significant heterogeneity and pleiotropy. Multivariate MR estimates indicated that there might be an interaction between E‐selectin and IL3Ra in the mechanism of WMPVS because their protective effect became insignificant after adjusting for each other. There were no significant eQTLs found in blood and brain tissues associated with the phenotype GWAS of WMPVS in SMR analysis. Instruments could also affect the phenotype by DNA methylation. According to the SMR analysis, we found that five DNAm probes in gene ABO were associated with the risk of WMPVS (*β*smr > 0, *P*smr < .05, FDR < .05). This suggests that low levels of E‐selectin and IL‐3Ra, mediated by related genetic variants (rs115478735, rs507666, rs2519093: intron variant), might increase the risk of WMPVS by elevating DNA methylation levels at the ABO gene locus.

PVS is a normal physiological anatomical structure between cerebral microvessels and the pia mater filled with cerebrospinal fluid. Its function is exchanging molecules such as glucose and lipids between cerebrospinal fluid and brain tissue and clearing metabolic byproducts like Aβ and Tau (Benveniste & Nedergaard, 2022; Hablitz et al., 2020; Mestre et al., 2017). The PVS would be visibly enlarged on the T2‐weighted MRI sequence when metabolic products accumulate abnormally (Wardlaw et al., 2020; Zhang et al., 2022). According to some clinical research, EPVS is considered one of the earliest markers of CSVD and is related to the development of WMH and CMBs (Benveniste & Nedergaard, 2022; Wardlaw et al., 2020). Therefore, exploring the underlying mechanisms of EPVS can be valuable in understanding the development of CSVD. E‐selectin, a surface glycoprotein molecule belonging to the cell adhesion molecule family, its expression can be increased by the stimulation from vascular endothelial cells and the binding of CD40L to CD40. E‐selectin plays a role in mediating the translocation of leukocytes to subendothelial tissues in inflammatory states caused by diseases such as arteriosclerosis, which is a marker of endothelial dysfunction (Motawi et al., 2012; Zhang et al., 2002). Previous cross‐sectional study revealed that high levels of serum E‐selectin were associated with the progression of CSVD (SVS, LI, CMB, and WMH), but no significant correlation was found between E‐selectin and EPVS (Zhang et al., 2022).

IL‐3Ra and IL‐5Ra are transmembrane glycoproteins that serve as receptors for the β common family cytokines IL‐3 and IL‐5, respectively. IL‐3Ra interacts with IL‐3 and mediates the involvement of IL‐3 in multiple biological processes. IL‐5Ra specifically binds IL‐5 to induce its signal transduction. Both IL‐3Ra and IL‐5Ra are involved in the pathogenesis of inflammatory diseases (Fogha et al., 2021; Kusano et al., 2012). There is currently limited research on the relationship between E‐selectin and IL‐3Ra, as well as IL‐3Ra and IL‐5Ra in neurological diseases. Our study found the possible association between these specific inflammatory proteins and WMPVS and elucidated this phenomenon at the genetic level. However, more research is required to validate our findings and explore the potential mechanism.

## 5 CONCLUSION

Based on the findings from MR and SMR analyses, we have discovered that upregulation of KNG1 gene expression in the frontal cortex could affect the risk associated with higher levels of TRAIL and SVS. Additionally, our study indicated a negative causal correlation between the levels of E‐selectin and IL‐3Ra and the risk of WMPVS, which might be mediated by the DNA methylation of the ABO gene in blood tissue.

### 5.1 Limitation

It should be noted that there were some limitations in this study. Previous studies on inflammation and CSVD have predominantly focused on common inflammatory factors, such as IL‐6, IL‐8, IL‐10, and others. As MR analysis relies on published genetic data, we encountered challenges in establishing a link between CSVD markers and these specific inflammatory factors due to inadequate quantity or linkage imbalances of instrumental variables. Furthermore, the conclusions drawn from our study require further validation through comprehensive experimental and clinical research to substantiate their applicability and relevance.

## AUTHOR CONTRIBUTIONS

**Tian‐Ci Qiao**: Methodology; writing—original draft. **Hao‐Yu Tian**: Writing—review and editing. **Shi‐Zhe Shan**: Validation. **Li‐Li Shan**: Data curation. **Zheng‐Yu Peng**: Data curation. **Jia Ke**: Investigation. **Meng‐Ting Li**: Resources; software. **Yang Wu**: Visualization. **Yan Han**: Conceptualization; writing—review and editing; funding acquisition.

### PEER REVIEW

The peer review history for this article is available at https://publons.com/publon/10.1002/brb3.3399.

## Supporting information

Supporting Information

Supporting Information

Table S1 Data source of exposure instruments (inflammatory related indicators).
Table S2 Data source of outcome instruments (CSVD markers).
Table S3 Significant estimate results of mendelian randomization studies.
Table S4 Details for significant SNP affecting inflammatory proteins levels and CSVD markers.
Table S5 eQTL probes identified in the SMR analysis for whole blood tissue.
Table S6 eQTL probes identified in the SMR analysis for brain tissue.
Table S7 mQTL probes identified in the SMR analysis for whole blood tissue.
Table S8 mQTL probes identified in the SMR analysis for brain tissue.

Figure S1 TNF‐related apoptosis‐inducing ligand (ebi‐a‐GCST90012011) on small vessel disease a. MR scatter plot, b. funnel plot, c. leave‐one‐out plot.
Figure S2 TNF‐related apoptosis‐inducing ligand (GCST90274843) on small vessel disease a. MR scatter plot, b. funnel plot, c. leave‐one‐out plot.
Figure S3 Interleukin‐1 receptor‐like 2 (ebi‐a‐GCST90019426) on small vessel disease a. MR scatter plot, b. funnel plot, c. leave‐one‐out plot.
Figure S4 *P*‐selectin glycoprotein ligand 1(ebi‐a‐GCST90012046) on small vessel disease a. MR scatter plot, b. funnel plot, c. leave‐one‐out plot.
Figure S5 TNF‐related apoptosis‐inducing ligand 1 (ebi‐a‐GCST90012011) on fractional anisotropy a. MR scatter plot, b. funnel plot, c. leave‐one‐out plot.
Figure S6 *E*‐selectin (ebi‐a‐GCST90012000) on cerebral microbleeds in any brain region a. MR scatter plot, b. funnel plot, c. leave‐one‐out plot.
Figure S7 Intercellular adhesion molecule 1 (prot‐a‐1397) on cerebral microbleeds in lobar brain region a. MR scatter plot, b. funnel plot, c. leave‐one‐out plot.
Figure S8 Interleukin‐22 receptor subunit alpha‐2 (prot‐a‐1511) on cerebral microbleeds in lobar brain region a. MR scatter plot, b. funnel plot, c. leave‐one‐out plot.
Figure S9 Interleukin‐18 (ebi‐a‐GCST90012024) on extensive basal ganglia perivascular space burden a. MR scatter plot, b. funnel plot, c. leave‐one‐out plot.
Figure S10 *E*‐Selectin (ebi‐a‐GCST90012000) on extensive basal ganglia perivascular space burden a. MR scatter plot, b. funnel plot, c. leave‐one‐out plot.
Figure S11 *E*‐Selectin (ebi‐a‐GCST90012000) on extensive white matter perivascular space burden a. MR scatter plot, b. funnel plot, c. leave‐one‐out plot.
Figure S12 Interleukin‐1 receptor‐like 2 (ebi‐a‐GCST90019426) on extensive white matter perivascular space burden a. MR scatter plot, b. funnel plot, c. leave‐one‐out plot.
Figure S13 Interleukin‐22 receptor subunit alpha‐2 (prot‐a‐1511) on extensive white matter perivascular space burden MR scatter plot, b. funnel plot, c. leave‐one‐out plot.
Figure S14 Interleukin‐3 receptor subunit alpha (prot‐a‐1530) on extensive white matter perivascular space burden MR scatter plot, b. funnel plot, c. leave‐one‐out plot.
Figure S15 Interleukin‐3 receptor subunit alpha (GCST90087655) on extensive white matter perivascular space burden a. MR scatter plot, b. funnel plot, c. leave‐one‐out plot.
Figure S16 Interleukin‐5 receptor subunit alpha (GCST90162097) on extensive white matter perivascular space burden MR scatter plot, b. funnel plot, c. leave‐one‐out plot.
Figure S17 Interleukin‐5 receptor subunit alpha (prot‐a‐1537) on extensive white matter perivascular space burden MR scatter plot, b. funnel plot, c. leave‐one‐out plot.

## Data Availability

All data relevant to this study are included in the supplementary files. These supplemental materials comprehensively provide the raw data and analytical results used in our research, facilitating verification, replication, or further analysis by other researchers. For additional information or inquiries, please contact the corresponding author of this paper.
