# Supplementary material for: Causal relationship between inflammatory factors and cerebral small vessel disease: Univariate, multivariate, and summary‐data‐based mendelian randomization analysis
Source: Brain Behav. 2024 Feb 10;14(2):e3399. doi: 10.1002/brb3.3399 (PMC10858724; doi:10.1002/brb3.3399)
Supplement: Supplementary file 4 — Figure S1 TNF‐related apoptosis‐inducing ligand (ebi‐a‐GCST90012011) on small vessel disease a. MR scatter plot, b. funnel plot, c. leave‐one‐out plot. Figure S2 TNF‐related apoptosis‐inducing ligand (GCST90274843) on small vessel disease a. MR scatter plot, b. funnel plot, c. leave‐one‐out plot. Figure S3 Interleukin‐1 receptor‐like 2 (ebi‐a‐GCST90019426) on small vessel disease a. MR scatter plot, b. funnel plot, c. leave‐one‐out plot. Figure S4 P‐selectin glycoprotein ligand 1(ebi‐a‐GCST90012046) on small vessel disease a. MR scatter plot, b. funnel plot, c. leave‐one‐out plot. Figure S5 TNF‐related apoptosis‐inducing ligand 1 (ebi‐a‐GCST90012011) on fractional anisotropy a. MR scatter plot, b. funnel plot, c. leave‐one‐out plot. Figure S6 E‐selectin (ebi‐a‐GCST90012000) on cerebral microbleeds in any brain region a. MR scatter plot, b. funnel plot, c. leave‐one‐out plot. Figure S7 Intercellular adhesion molecule 1 (prot‐a‐1397) on cerebral microbleeds in lobar brain region a. MR scatter plot, b. funnel plot, c. leave‐one‐out plot. Figure S8 Interleukin‐22 receptor subunit alpha‐2 (prot‐a‐1511) on cerebral microbleeds in lobar brain region a. MR scatter plot, b. funnel plot, c. leave‐one‐out plot. Figure S9 Interleukin‐18 (ebi‐a‐GCST90012024) on extensive basal ganglia perivascular space burden a. MR scatter plot, b. funnel plot, c. leave‐one‐out plot. Figure S10 E‐Selectin (ebi‐a‐GCST90012000) on extensive basal ganglia perivascular space burden a. MR scatter plot, b. funnel plot, c. leave‐one‐out plot. Figure S11 E‐Selectin (ebi‐a‐GCST90012000) on extensive white matter perivascular space burden a. MR scatter plot, b. funnel plot, c. leave‐one‐out plot. Figure S12 Interleukin‐1 receptor‐like 2 (ebi‐a‐GCST90019426) on extensive white matter perivascular space burden a. MR scatter plot, b. funnel plot, c. leave‐one‐out plot. Figure S13 Interleukin‐22 receptor subunit alpha‐2 (prot‐a‐1511) on extensive white matter perivascular space burden MR scatter plot, b. fun [file BRB3-14-e3399-s001.docx]

a. b. c.


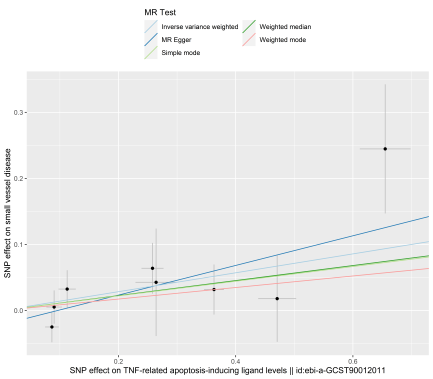

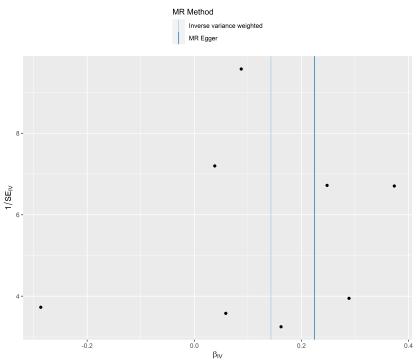

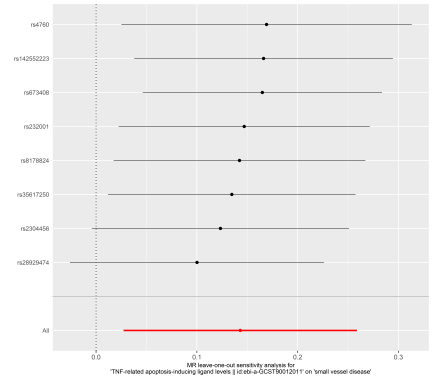


Figure S1. TNF-related apoptosis-inducing ligand (ebi-a-GCST90012011) on small vessel disease

a. MR scatter plot, b. funnel plot, c. leave-one-out plot

a. b. c.


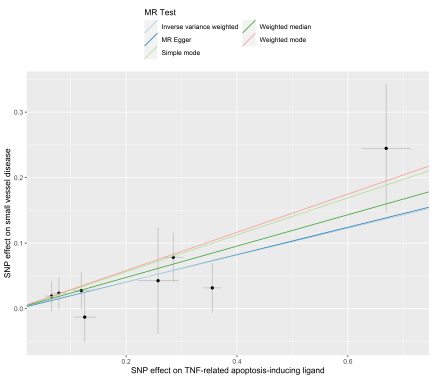

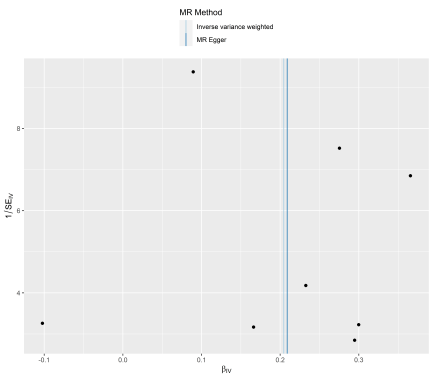

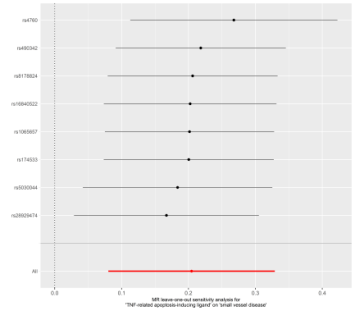


Figure S2. TNF-related apoptosis-inducing ligand (GCST90274843) on small vessel disease

a. MR scatter plot, b. funnel plot, c. leave-one-out plot

a. b. c.


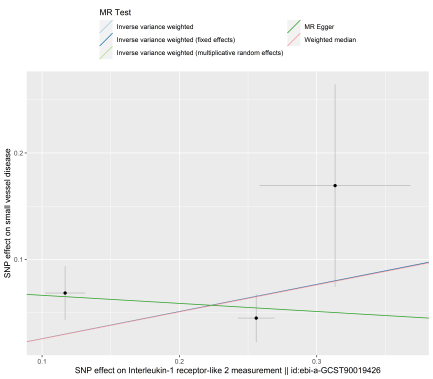

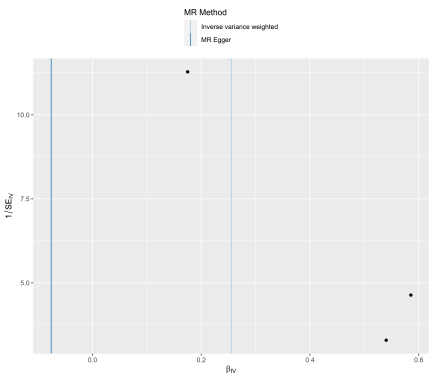

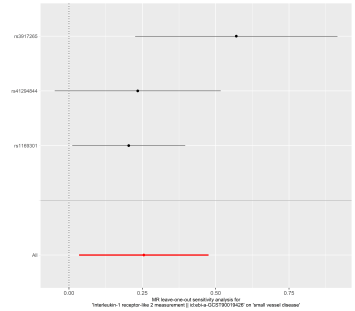


Figure S3. Interleukin-1 receptor-like 2 (ebi-a-GCST90019426) on small vessel disease

a. MR scatter plot, b. funnel plot, c. leave-one-out plot

a. b. c.


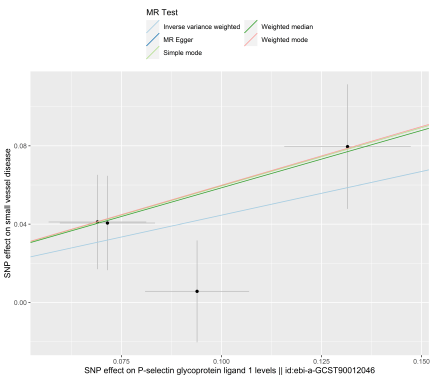

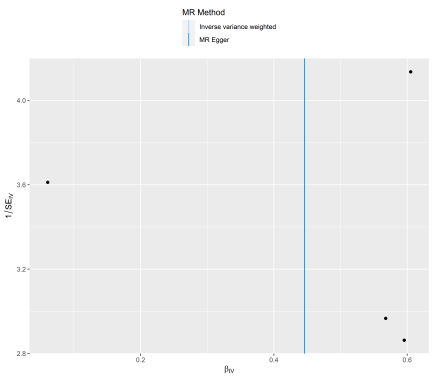

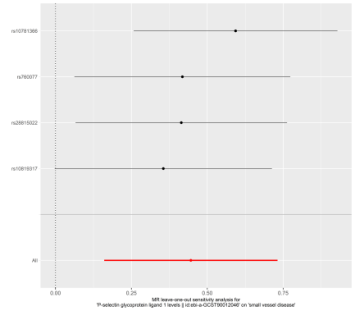


Figure S4. P-selectin glycoprotein ligand 1(ebi-a-GCST90012046) on small vessel disease

a. MR scatter plot, b. funnel plot, c. leave-one-out plot

a. b. c.


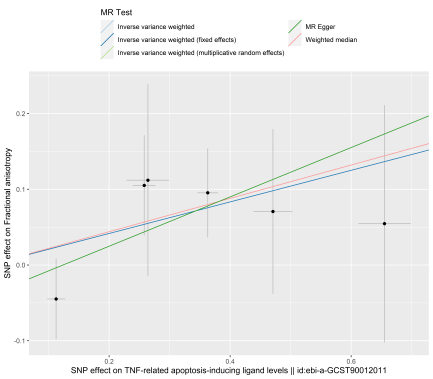

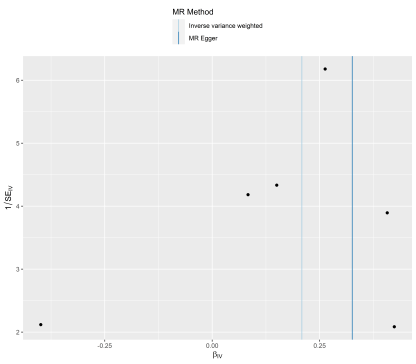

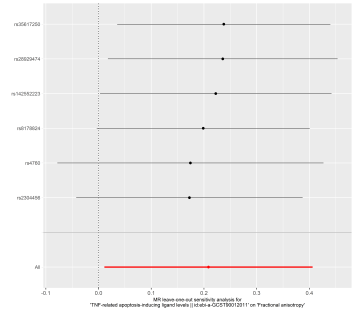


Figure S5. TNF-related apoptosis-inducing ligand 1 (ebi-a-GCST90012011)on fractional anisotropy

a. MR scatter plot, b. funnel plot, c. leave-one-out plot

a. b. c.


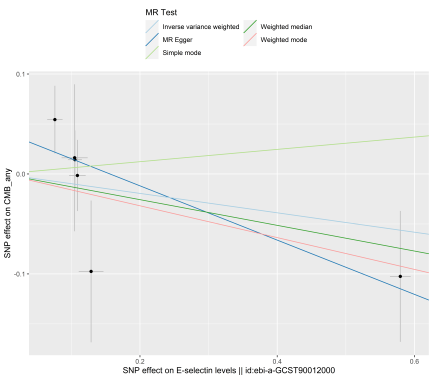

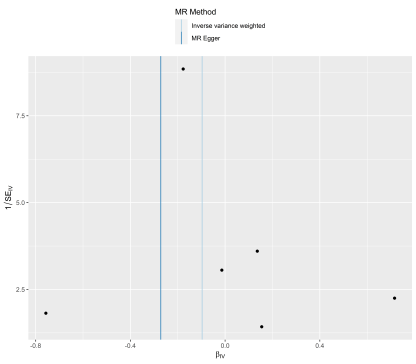

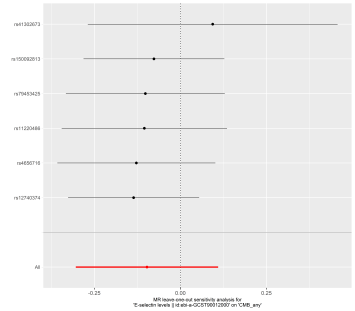


Figure S6. E-selectin (ebi-a-GCST90012000) on cerebral microbleeds in any brain region

a. MR scatter plot, b. funnel plot, c. leave-one-out plot

a. b. c.


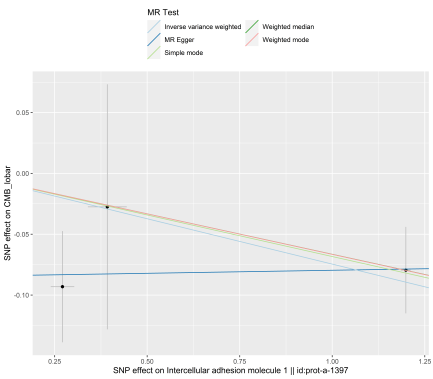

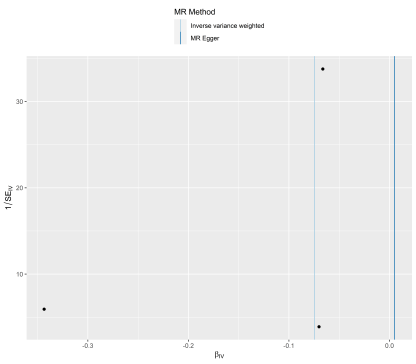

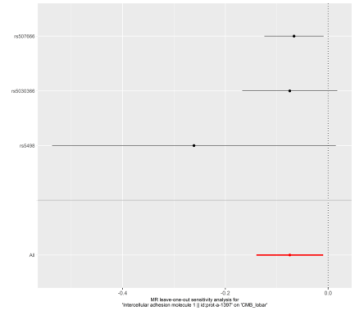


Figure S7. Intercellular adhesion molecule 1 (prot-a-1397) on cerebral microbleeds in lobar brain region

a. MR scatter plot, b. funnel plot, c. leave-one-out plot

a. b. c.


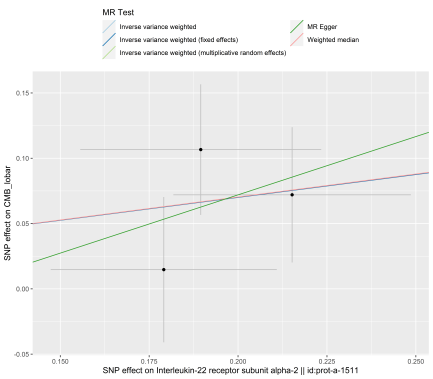

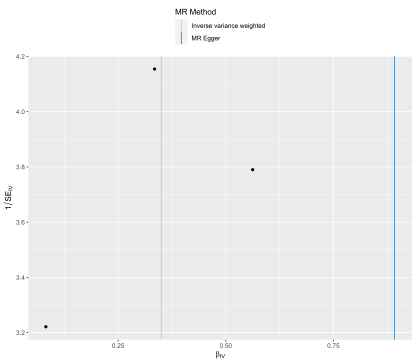

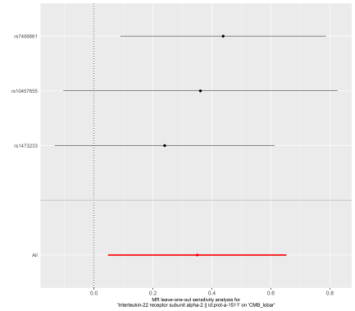


Figure S8. Interleukin-22 receptor subunit alpha-2 (prot-a-1511) on cerebral microbleeds in lobar brain region

a. MR scatter plot, b. funnel plot, c. leave-one-out plot

a. b. c.


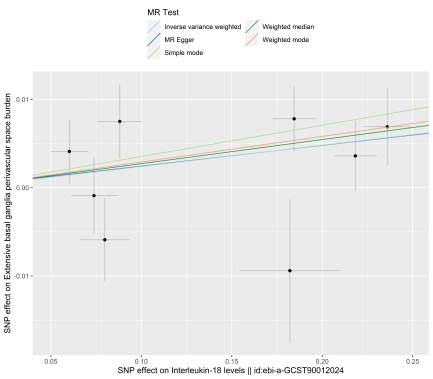

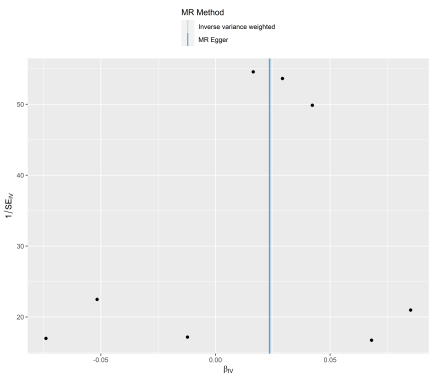

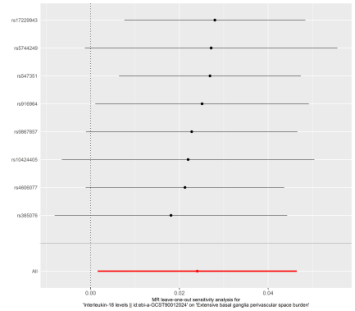


Figure S9. Interleukin-18 (ebi-a-GCST90012024) on extensive basal ganglia perivascular space burden

a. MR scatter plot, b. funnel plot, c. leave-one-out plot

a. b. c.


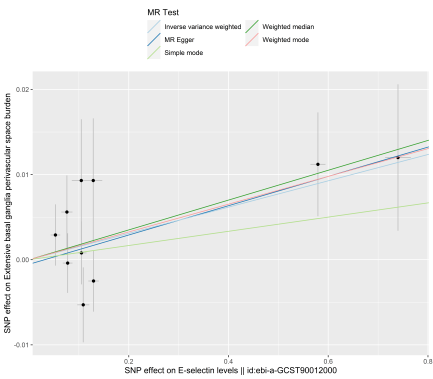

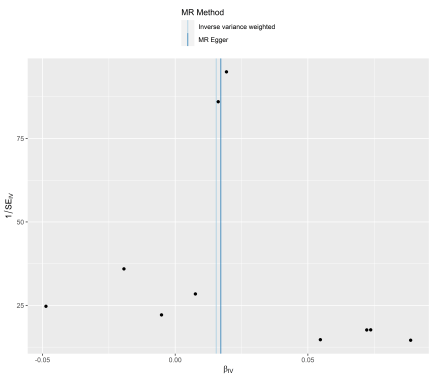

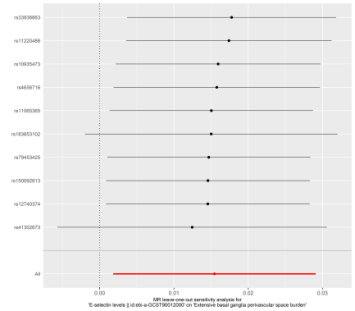


Figure S10. E-selectin (ebi-a-GCST90012000) on extensive basal ganglia perivascular space burden

a. MR scatter plot, b. funnel plot, c. leave-one-out plot

a. b. c.


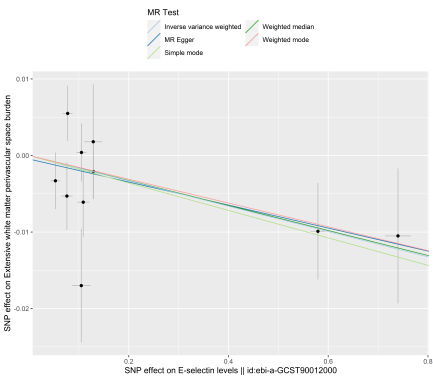

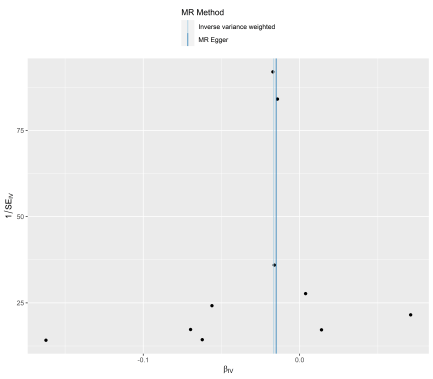

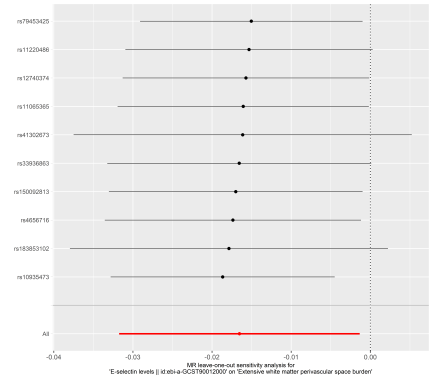


Figure S11. E-selectin (ebi-a-GCST90012000) on extensive white matter perivascular space burden

a. MR scatter plot, b. funnel plot, c. leave-one-out plot

a. b. c.


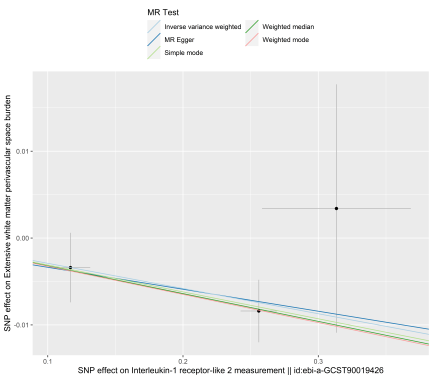

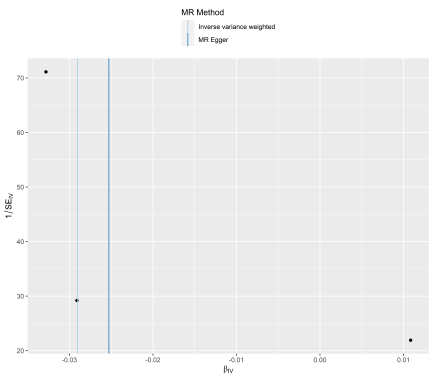

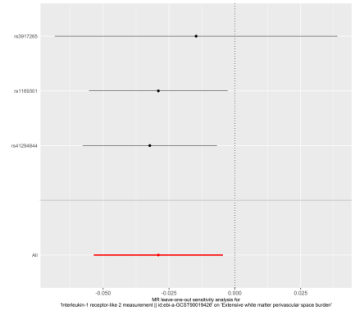


Figure S12. Interleukin-1 receptor-like 2 (ebi-a-GCST90019426) on extensive white matter perivascular space burden

a. MR scatter plot, b. funnel plot, c. leave-one-out plot

a. b. c.


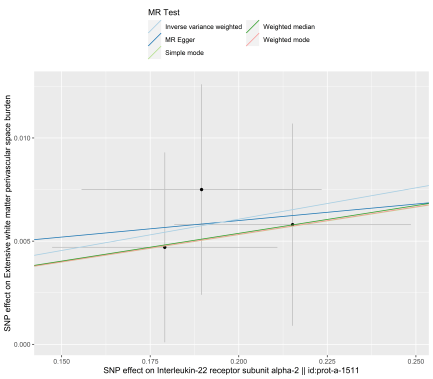

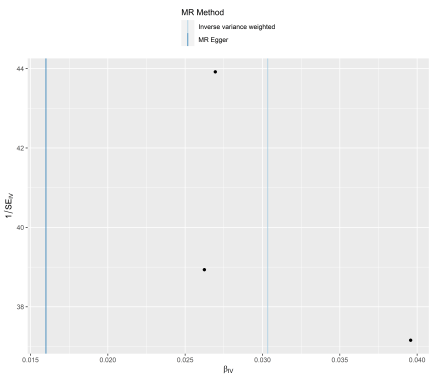

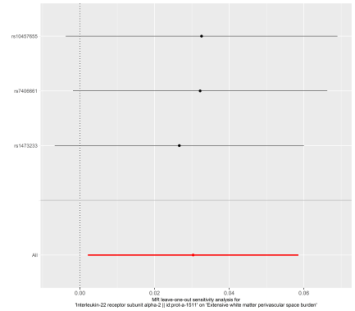


Figure S13. Interleukin-22 receptor subunit alpha-2 (prot-a-1511) on extensive white matter perivascular space burden

1. MR scatter plot, b. funnel plot, c. leave-one-out plot

a. b. c.


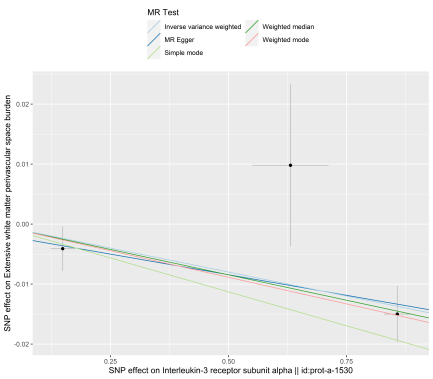

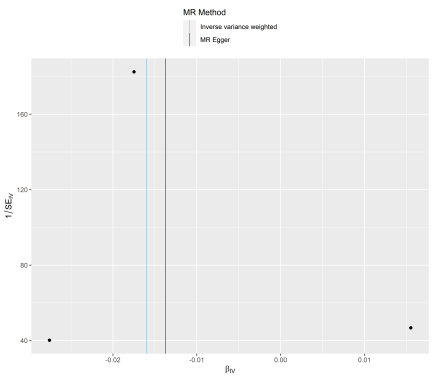

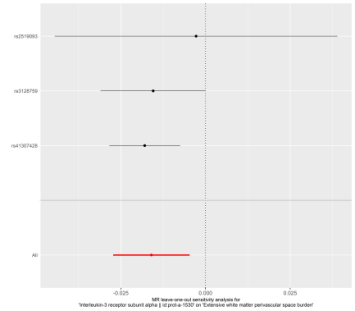


Figure S14. Interleukin-3 receptor subunit alpha (prot-a-1530)on extensive white matter perivascular space burden

1. MR scatter plot, b. funnel plot, c. leave-one-out plot

a. b. c.


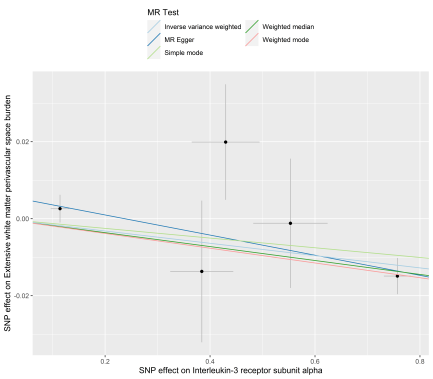

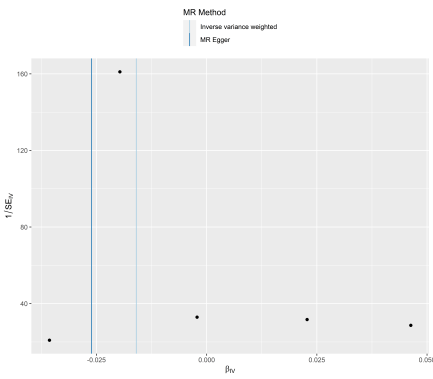

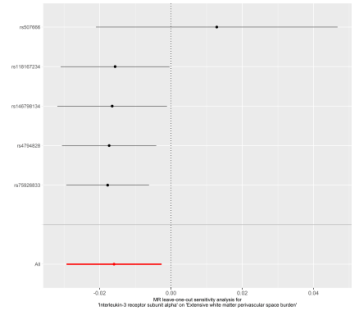


Figure S15. Interleukin-3 receptor subunit alpha (GCST90087655) on extensive white matter perivascular space burden

a. MR scatter plot, b. funnel plot, c. leave-one-out plot

a. b. c.


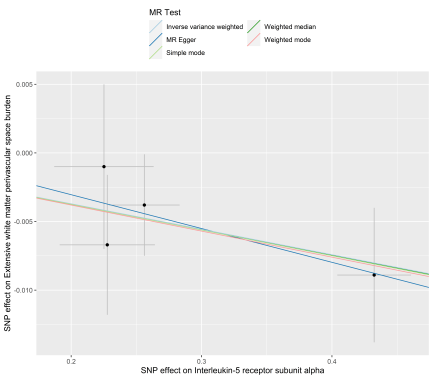

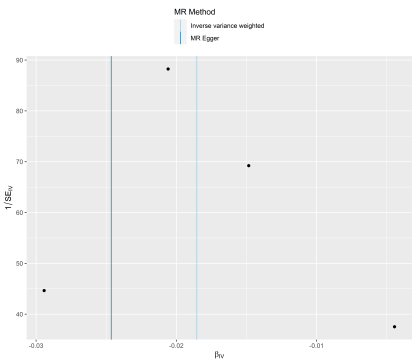

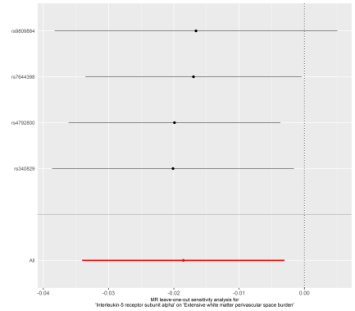


Figure S16. Interleukin-5 receptor subunit alpha (GCST90162097) on extensive white matter perivascular space burden

1. MR scatter plot, b. funnel plot, c. leave-one-out plot

a. b. c.


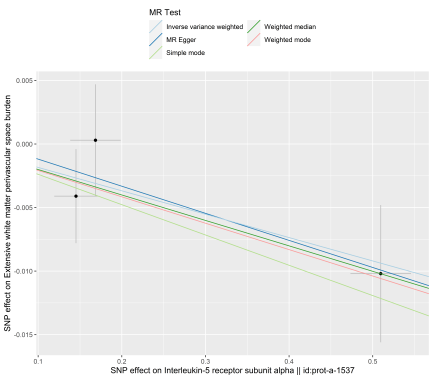

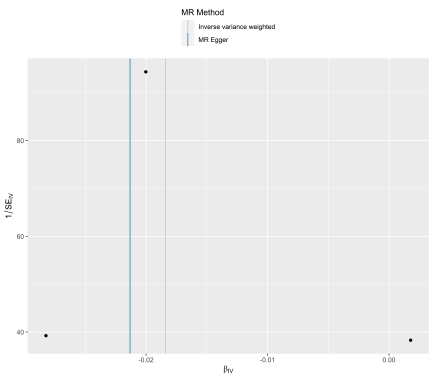

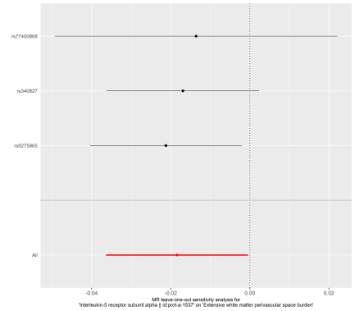


Figure S17. Interleukin-5 receptor subunit alpha (prot-a-1537) on extensive white matter perivascular space burden

1. MR scatter plot, b. funnel plot, c. leave-one-out plot
